# Shape and position of the node and notochord along the bilateral plane of symmetry are regulated by cell–extracellular matrix interactions

**DOI:** 10.1242/bio.20148243

**Published:** 2014-06-13

**Authors:** Maria Pulina, Dong Liang, Sophie Astrof

**Affiliations:** Department of Medicine, Center for Translational Medicine, Thomas Jefferson University, Philadelphia, PA 19107, USA; * Present address: Laboratory of Molecular and Cellular Neuroscience, Rockefeller University, New York, NY 10065, USA.

**Keywords:** Fibronectin, Integrin alpha 5, Extracellular matrix, Node, Notochord, Bilateral symmetry

## Abstract

The node and notochord (and their equivalents in other species) are essential signaling centers, positioned along the plane of bilateral symmetry in developing vertebrate embryos. However, genes and mechanisms regulating morphogenesis of these structures and their placement along the embryonic midline are not well understood. In this work, we provide the first evidence that the position of the node and the notochord along the bilateral plane of symmetry are under genetic control and are regulated by integrin α5β1 and fibronectin in mice. We found that the shape of the node is often inverted in integrin α5-null and fibronectin-null mutants, and that the positioning of node and the notochord is often skewed away from the perceived plane of embryonic bilateral of symmetry. Our studies also show that the shape and position of the notochord are dependent on the shape and embryonic placement of the node. Our studies suggest that fibronectin regulates the shape of the node by affecting apico-basal polarity of the nodal cells. Taken together, our data indicate that cell–extracellular matrix interactions mediated by integrin α5β1 and fibronectin regulate the geometry of the node as well as the placement of the node and notochord along the plane of bilateral symmetry in the mammalian embryo.

## INTRODUCTION

Development of the embryonic midline structures is an essential process during embryogenesis, yet it remains poorly understood (Mikawa et al., 2004; Stemple, 2005). One of the earliest known events leading to the establishment of the embryonic midline is induction of the primitive streak through the expression of Vg1 by cells of the posterior marginal zone in chick embryos (Wei and Mikawa, 2000). The primitive streak in the chick and mouse is the first morphological structure marking the plane of bilateral symmetry in the developing embryo. The anterior part of the primitive streak is molecularly distinct from the posterior primitive streak and gives rise to two other essential midline structures, the node and the notochord (Lee and Anderson, 2008).

Proper development of the node and the notochord are requisite for the ensuing vertebrate development (Meno et al., 1998; Stemple, 2005; Shiratori and Hamada, 2006). These structures are required for the establishment and maintenance of the left–right embryonic axis, which, in turn, is essential for the development and proper function of all visceral organs (Burdine and Schier, 2000; Tabin, 2006; Yashiro et al., 2007; Lee and Anderson, 2008). In addition, the notochord provides essential structural and organizing functions during development; factors synthesized by the notochord are essential for the establishment of the dorso-ventral polarity of the neural tube, patterning of the definitive endoderm, and development of the dorsal aorta and the heart, among others (Fouquet et al., 1997; Sumoy et al., 1997; Goldstein and Fishman, 1998; Stemple, 2005; Bressan et al., 2009). Defective development and differentiation of the node and the notochord give rise to severe embryonic defects in model organisms and cause birth defects in humans (Almog et al., 2001; Ramsdell, 2005; Turgut et al., 2006).

Embryonic development involves finely orchestrated interactions among cells and tissues derived from every germ layer. Thus the position, shape and function of each organ and structure are important and ultimately depend on interactions of cells with their extracellular microenvironment. For example, deletion of laminin γ1, a component of 10 laminin heterotrimers (Yurchenco, 2011), leads to embryonic demise before gastrulation due to defective formation of the Reichert's membrane (Miner and Yurchenco, 2004); deletion of integrin β1 chain, a component of the twelve known integrin heterodimers is lethal before implantation (Wickström et al., 2011). Similarly, absence of cytoplasmic mediators propagating signals from the ECM such as talins or kindlins, also lead to early embryonic lethality (Wickström et al., 2011). Interestingly, in the examples described above, the main causes of aberrant embryonic morphogenesis, even at the earliest stages, are thought to be defective establishment and/or maintenance of cell polarity.

Integrins are a major class of transmembrane proteins connecting the extracellular matrix (ECM) proteins with the cytoskeletal machinery regulating cellular responses to ECM (Hynes, 2002; Whittaker and Hynes, 2002; Whittaker et al., 2006). Integrins are heterodimers composed of one alpha and one beta subunits, both chains are type-I transmembrane proteins. There are 18 known integrin alpha chains and 8 known integrin beta chains in mammals giving rise to twenty-four different integrin heterodimers with unique and overlapping specificities for their extracellular ligands (Huttenlocher and Horwitz, 2011). Integrin α5 is known to exclusively heterodimerize with integrin β1 (Hynes, 2002); the integrin α5β1 heterodimer is a major receptor for fibronectin (FN1), a large ECM protein essential for embryonic development and adult homeostasis (Hynes, 2009; Hynes, 2012). In vitro experiments demonstrated that the binding of α5β1 to FN1 leads to activation of extracellular signal-regulated kinases (Erks) and other intracellular signal mediators regulating cell migration, survival and proliferation (Schwartz, 2010; Geiger and Yamada, 2011; Huttenlocher and Horwitz, 2011; Wickström et al., 2011). Other cell-surface integrins can also function as FN1 receptors, and include αv-containing integrin heterodimers, as well as integrins α4β1, α8β1, and α9β1 (Wickström et al., 2011). However, when compared with the deletion of any other integrin alpha chain, genetic ablation of integrin α5 leads to the most severe embryonic phenotype, which is also similar to the phenotype of FN1-null embryos (George et al., 1993; Yang et al., 1993; George and Hynes, 1994; George et al., 1997; Takahashi et al., 2007). Consistent with in vitro studies, mouse mutants expressing a defective form of FN1, in which α5β1 binding motif Arg–Gly–Asp is mutated, develop phenotypes comparable with those of integrin α5-null embryos (Takahashi et al., 2007). Studies from our own lab demonstrated that integrin α5β1 and FN1 play similar roles in the development of the cardiac neural crest, formation of the heart and in establishment and maintenance of the left–right body plan (Mittal et al., 2010; Pulina et al., 2011; Mittal et al., 2013). Taken together, these data indicate that in early embryogenesis, integrin α5β1 is a major modulator of signaling by FN1 and that cell–ECM interactions mediated by the binding of integrin α5β1 to FN1 play essential roles in embryonic development.

In this paper we report our novel findings that integrin α5β1 and its ligand FN1 are essential for the development and the placement of the node and the notochord along the embryonic plane of bilateral symmetry. We also show that the proper geometry of the node is essential for the proper shape and placement of the notochord relative to the embryonic plane of bilateral symmetry.

## MATERIALS AND METHODS

### Animal models

*FN1* and *integrin α5* null mutations were generated by the Richard Hynes lab (Yang et al., 1993; George et al., 1997) and are available from the Jackson labs. FN1-null or integrin α5-null embryos were obtained by mating heterozygous adult mice of C57BL/6J genetic background. All experiments involving vertebrate animals were approved by the Institutional Animal Care and Use Committee of Weill Cornell Medical School and Thomas Jefferson University, and were performed in accordance with federal guidelines for humane care of animals.

### Microscopy

Scanning electron microscopy, in situ hybridization and confocal fluorescence microscopy were performed as described (Pulina et al., 2011). For scanning electron microscopy, embryos were collected in the evening of embryonic day (E) 7.5. For immunofluorescence and in situ hybridization experiments, embryos were collected at E8.0 and ranged from the late headfold stage, as defined by Downs and Davies to the 4 somite stage (Downs and Davies, 1993). We used Imaris software (Bitplane) to perform 3D reconstructions of confocal data and for data analysis. Controls and mutants were stained simultaneously using the same solutions, in the same Eppendorf tubes or glass vials, and imaged on the same days using identical microscope settings. The images were then manipulated utilizing Imaris software, using identical settings for manipulation of image brightness and background. When integrin α5-null mutants were analyzed, their wild-type or heterozygous littermates served as controls. Similarly, when FN1-null mutants were analyzed, their wild-type or heterozygous littermates were used as controls. All embryos were genotyped by using yolk sacs as described (Goh et al., 1997; Astrof et al., 2007).

## RESULTS AND DISCUSSION

### Integrin α5β1 regulates the shape and position of the node and notochord

**Fig. 1.**
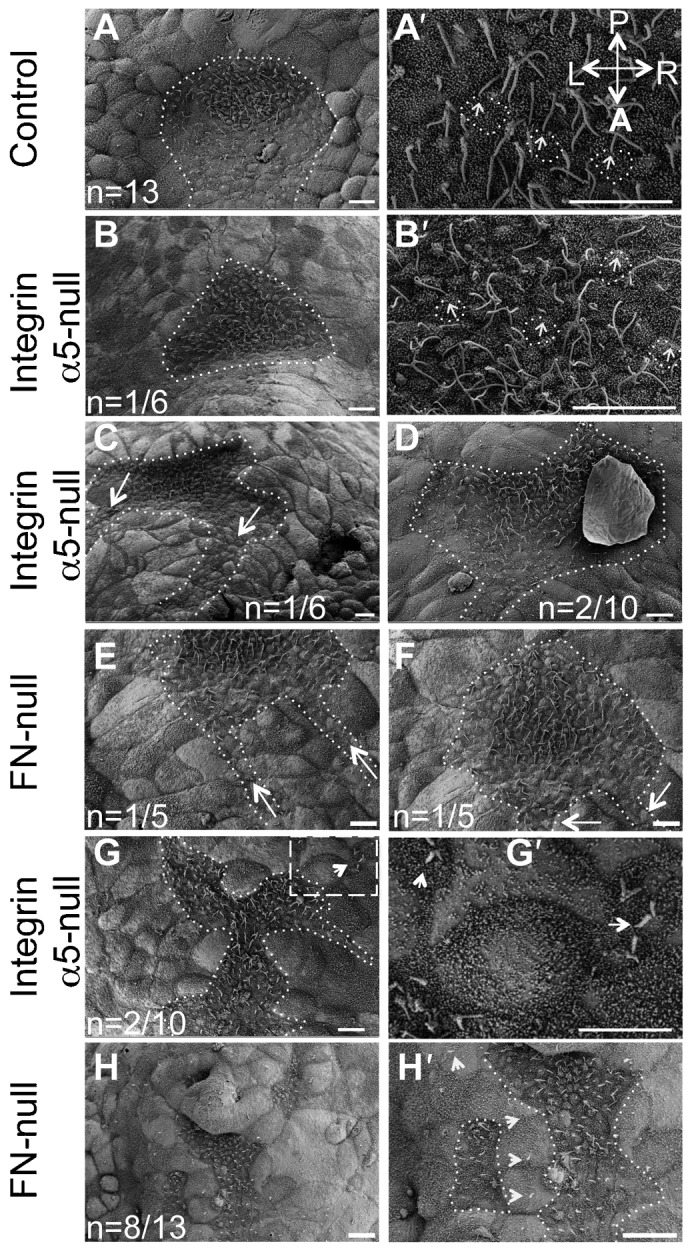
Integrin α5 and fibronectin are required for morphogenesis of the mouse node. (A,A′) Shape of the node in the control mouse embryo at E7.5. (A) The dotted line outlines the entire shape of the node. (A′) Primary cilia (arrows) are positioned at the posterior of nodal cells (some examples are outlined). (B,B′,C,D,G,G′) Node shapes are aberrant in integrin α5-null E7.5 embryos. (B) Notice the inverted shape of the node in this integrin α5-null mutant. Nodes were inverted in 6 out of 10 integrin α5-null mutants. (B,C) Examples of such inverted nodes. (B′) Magnified view of cilia in the node shown in panel B. Each primary cilium (arrows) is positioned at the posterior of each cell (outlined). (C) Note two notochords emanating from each corner of a mutant node (dotted line, arrows). (D) The pointy end of the node was positioned in a correct orientation in 2/10 integrin α5-null mutant nodes (dotted line outlines the node and the notochord). (E,F,H,H′) Aberrant nodes in E7.5 FN1-null mutants. (E,F) Two examples of inverted nodes in FN1-null mutants. Nodes were inverted in 5 out of 13 FN1-null mutants. (G,H) In some α5-null and FN1-null mutants, the nodes were narrow and disrupted. (G′,H′) Magnified views of panels G and H. Areas containing node cells are outlined. Arrows point to cilia protruding from beneath the cells of the primitive endoderm. A–anterior, P–posterior, L–left and R–right axes are marked and their directions are the same in all panels. Scale bars: 10 µm.

Our earlier studies demonstrated that integrin α5 regulates the development of the left–right axis of asymmetry (Pulina et al., 2011). In order to determine the function of integrin α5 during left–right axis formation, we examined formation of the mouse node in integrin α5-null mutants using scanning electron microscopy (SEM) and found that the node is formed aberrantly in all examined mutants, n = 10 (Fig. 1; Table 1). In control embryos, the nodes were always oriented such that the pointy, narrow end of the node was facing the anterior of the embryo (Fig. 1A). The node was invaginated with a characteristic “tear-drop shape”, and was aligned with the bilateral plane of embryonic symmetry (Lee and Anderson, 2008). However, nodes in all integrin α5-null embryos were flattened and aberrant in shape (Fig. 1B,C,G). In 2 out of 10 integrin α5-null mutant embryos, nodes were oriented correctly, with the narrow end pointing in the anterior direction (Fig. 1D), while in 6 out of 10 integrin α5-null mutants, nodes were inverted in shape, such that the narrow end of the node was facing the posterior instead of the anterior of the embryo (Table 1). We define the “inverted node phenotype” as that of a flipped triangle, in which the narrow end is facing the posterior, while the wide side is facing the anterior of the embryo (schematic inset in Figs 4, 5). This is a 180° rotation along the left–right axis of the embryos. Fig. 1B,C shows 2 examples of such nodes.

**Table 1.**
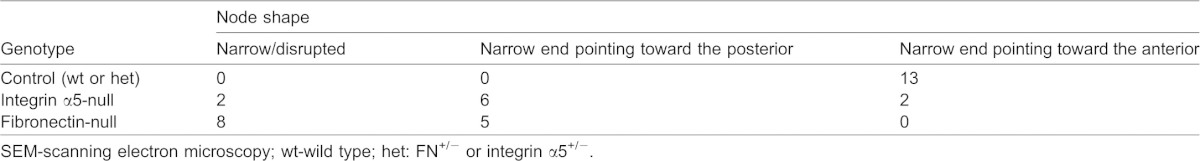
Summary of node shapes seen with SEM

**Fig. 2.**
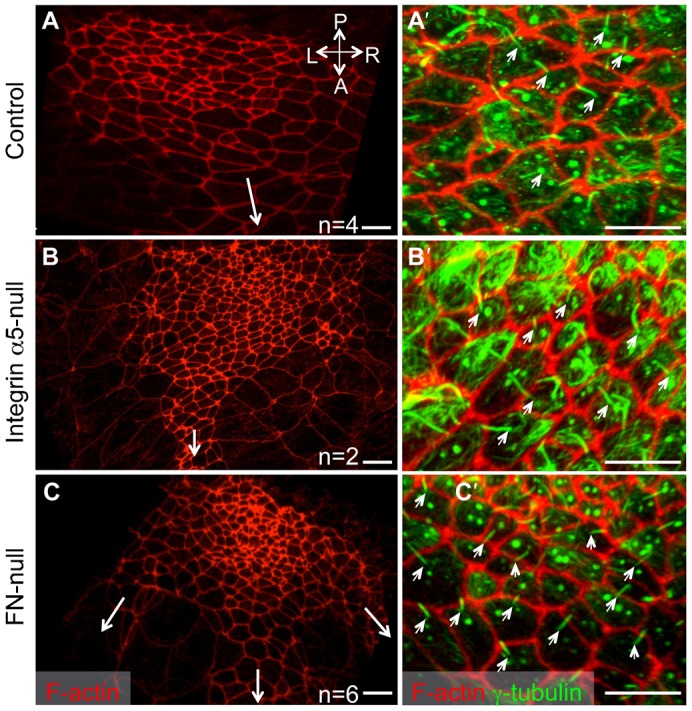
Anterior–posterior polarity of the nodal cells does not depend on integrin α5β1 or FN1. Red: F-actin is detected using rhodamine-phalloidin; Green: acetylated (stable) α-tubulin. Positions of the notochords are marked by long arrows in panels A–C. Note three notochords originate from the middle and the two corners of the inverted node in this FN1-null embryo (arrows, C). Protruding cilia (small arrows, A′–C′) are located at the posterior of each nodal cell. Axes are marked as in Fig. 1. All embryos were collected in the morning of E8.0. Scale bars: 10 µm.

Interestingly, despite the apparent shape inversion, the anterior–posterior specification of the node and the cells within the node was preserved. For example, the cilia emanating from the ventral surface of these inverted nodes were positioned toward the posterior region of nodal cells (Fig. 1A′,B′, Fig. 2), and the presumptive notochordal cells were always seen coming out of the anterior-facing side of the node (Figs 1, 2, 4, 5, 6). Thus, while integrin α5 is required to generate the proper geometry of the node, it is not essential for the establishment of the anterior–posterior embryonic or cellular axis. These findings are consistent with prior studies demonstrating that the node is not essential for establishing anterior–posterior polarity of the developing mouse embryo (Davidson et al., 1999).

Other examined integrin α5-mutants (n = 2 of 10) had narrow, disrupted nodes (Fig. 1G) with some of the node cells located underneath the large cells of the visceral endoderm (Fig. 1G,G′). This suggests that integrin α5 may play a role in the process of intercalation, during which mesendodermal cells of the midline push the cells of the visceral endoderm apart and appear on the embryonic surface (Lee and Anderson, 2008). This process of intercalation is also important for the development of the definitive endoderm (Kwon et al., 2008), and indeed, we found that the gut in integrin α5-null embryos or FN1-null embryos did not form properly (Villegas et al., 2013). Two out of ten integrin α5-null mutants examined by SEM had a node with the narrow end positioned in a proper anterior–posterior orientation (Fig. 1D). Unlike the control, the nodal pits in these mutants were shallow and irregular in shape.

The 3D shape of the mouse node is thought to be important for the establishment of the left–right body plan (Cartwright et al., 2004), and mouse mutants, in which the shape of the node is altered exhibit randomized left–right body plan (Lee and Anderson, 2008; Lee et al., 2010). Thus defective establishment of the left–right axis in integrin α5-null mutants (Pulina et al., 2011) can be explained by aberrant morphogenesis of the node. Taken together, our studies indicate that integrin α5β1 plays an important role in embryo development by regulating node morphogenesis and the establishment of the left–right body plan.

### Fibronectin regulates the geometry and midline placement of the node and notochord

FN1 is a major extracellular ligand for the integrin α5β1 (Hynes, 2012). If integrin α5β1 regulates formation of the stereotypical shape of the mouse node by binding FN1, we would expect to find shallow, inverted and disrupted nodes in FN1-null mutants as well. Indeed, our SEM studies showed that in 5 out of 13 FN1-null mutants had inverted nodes and 8 out of 13 FN1-null mutants had narrow and discontinuous nodes (Fig. 1E,F,H,H′), consistent with our previous study (Pulina et al., 2011). We found that, like in integrin α5-null mutants, the presumptive notochordal cells originated from either corner of the anterior-facing mutant node (Fig. 1E,F, two different examples of mutant nodes are shown). Similar to integrin α5-null mutants, the primary cilia on the ventral cells of FN1-null nodes were properly positioned toward the cells' posterior (Fig. 2C,C′). These findings suggest that FN1 and its major integrin receptor α5β1 regulate geometry of the node but not the overall anterior–posterior polarity of the embryo or of the cells composing the node.

### Fibronectin regulates apico-basal polarity of nodal cells

**Fig. 3.**
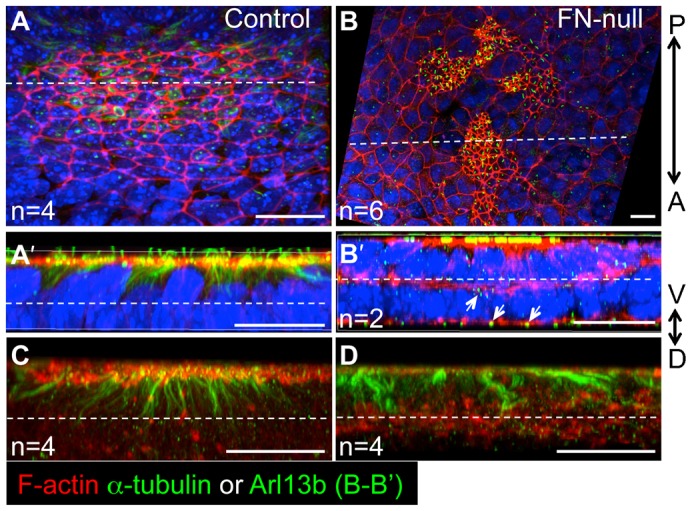
FN1 is required for the apico-basal polarity of the nodal cells. Transverse optical sections through the node (dotted lines in panels A and B indicate the plane of optical section shown in panels A′ and B′). 3D reconstructions of views through the transverse edge of each section are presented. In each view (A′,B′), multiple layers of cells posterior to the dotted lines in panels A and B are collapsed into one plane. F-actin (red) and acetylated α-tubulin and cilia (green) are only present at the apical (ventral) surface of the ventral-most nodal cells in controls (A′,C). The dotted lines in panels A′,B′,C and D mark 10 µm from the apical surface of the ventral node, and indicate about one cell layer. In FN1-null mutants (B,B′,D), F-actin is distributed apically and basally and its presence is not restricted to the ventral cell surface. In FN1-null embryos, cilia, visualized using anti-Arl13b antibody (B′) or anti-acetylated α-tubulin antibody (D), are present on ventral and internal cells of the node, and point apically and basally (B′, arrows). (C,D) Embryos were stained to detect F-actin and acetylated α-tubulin. The views are 3D reconstructions through multiple cell layers of the nodes as described in panels A′ and B′. The absence of nuclear stain allows visualizing the distribution of F-actin and α-tubulin through the nodes, internally. F-actin is localized apically in all cells of the node, while in mutants F-actin is re-distributed from the apical surface throughout the cells in the mutants nodes. Axes are as in Fig. 2. All embryos were collected in the morning of E8.0. Scale bars: 20 µm.

We hypothesized that defective node morphogenesis in FN1-null mutants was caused by defective apico-basal polarity of the cells composing the node. To test this idea, we stained control and mutant embryos using rhodamine-conjugated phalloidin to detect F-actin. In addition, control and FN1-null embryos were co-stained using antibodies to acetylated α-tubulin (components of stable microtubules, including those inside the primary cilia) or antibodies to the ciliary protein Arl13b, which localizes to primary cilia (Caspary et al., 2007). The ventral-most cells of the wild-type node are apically constricted and assemble F-actin rings at the constricted, ventral-most surfaces of nodal cells (Fig. 3A,A′) (Lee and Anderson, 2008). Another notable feature of the properly apically polarized cells of the wild-type node is the presence of stable microtubules and the primary cilia at the apical side of the ventral-most cells of the node (Lee and Anderson, 2008). These two features of apico-basal polarity were disrupted in FN1-null mutants (Fig. 3B,B′,D). Remarkably, primary cilia marked by the expression of Arl13B were found both at apical and basal surfaces of nodal cells in FN1-null mutants (Fig. 3B′, arrows). Future experiments utilizing additional cell polarity markers and earlier time points during nodal morphogenesis are necessary to further confirm the polarity status of nodal cells in the mutants and to determine whether FN1 regulates the establishment or the maintenance of nodal cell polarity. Upon examination of integrin α5-null nodes, we found ectopical distribution of F-actin in one out of three mutant embryos (data not shown), suggesting that additional FN1-binding integrins regulate apico-basal polarity of the nodal cells.

### Integrin α5β1 and fibronectin are required to position the node and notochord along the plane of mirror symmetry in developing mouse embryos

At E7.5–E8.0, wild-type embryos appear bilaterally symmetrical and have no external, gross morphological features that distinguish the left side from the right. The primitive streak and the node are bilaterally symmetrical and lie along the bilateral plane of symmetry. Cells of the notochord arise from the node (Yamanaka et al., 2007), and the narrow part of the node and the notochordal plate align along the plane of bilateral symmetry in wild-type embryos, suggesting that the shape of the node and the positioning of the narrow part of the node along the midline could be important for the placement of the narrow strip of notochordal cells along the embryonic plane of bilateral symmetry.

**Fig. 4.**
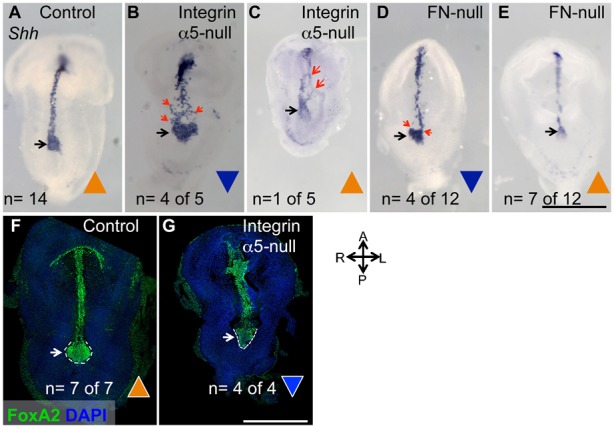
Integrin α5 and FN1 are required the proper geometry of the node and the morphogenesis of the notochord as shown by the expression of *Shh* and FoxA2. (A–E) Whole-mount in situ hybridizations using a mouse Shh anti-sense RNA probe. In controls (A) the narrow tip of the node points anteriorly and is contiguous with the narrow strip of the notochord. In mutants with inverted nodes, notochordal cells emanate from both corners of the node (B), giving rise to a dispersed notochord (red arrows). (C) Notochordal cells in this mutant “follow” the bends of the neural tube (red arrows). (D) When most notochordal cells emanate from one corner or (E) from a narrow tip of the node (black arrows), the notochords are narrow in the mutants. Triangles indicate properly oriented (orange) or inverted nodes (blue). (F,G) FoxA2 protein expression (green). 3D reconstructions of immunofluorescence confocal images of control (F) and integrin α5-null embryos (G), demonstrating inverted nodes in the mutants. Embryos were collected at E8.0. Littermate control embryos ranged from 2–5 somites. Mutants do not develop somites. Axes are labeled. Scale bars: 500 µm.

**Fig. 5.**
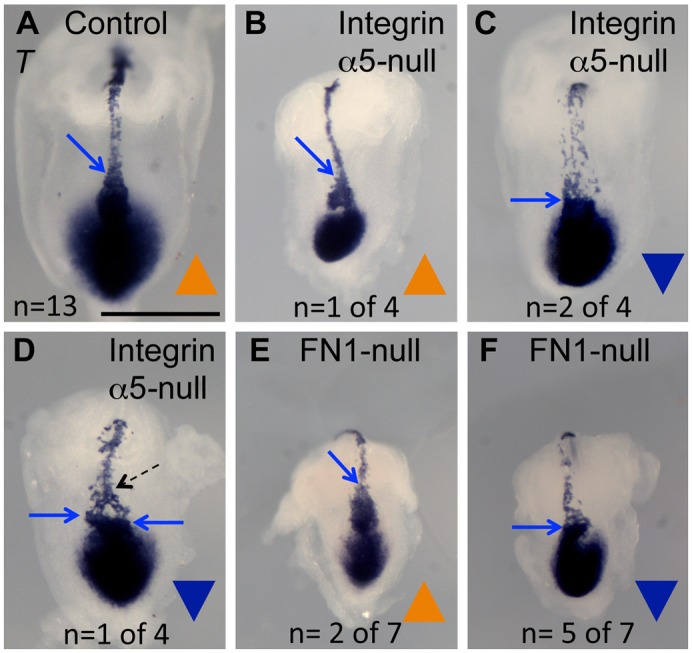
Integrin α5 and FN1 regulate the geometry of the node and the development of the notochord as shown by the expression of *T*. Whole-mount in situ hybridizations using an anti-sense *T* RNA probe. (A) Control. (B–D) Integrin α5-null mutants. (E,F) FN1-null mutants. In those mutants in which the node is oriented with the pointy end toward the anterior (blue arrows, B,E) and along the midline, the notochords are positioned at the embryonic midline and are narrow (B). In mutants with inverted nodes, notochordal cells emanate from the entire wide face of the node (blue arrow), giving rise to the wide notochord (C,F), or from two corners of the node (D). (D) Notochordal cells are refocused along the midline anteriorly (dotted arrow). Embryos were collected at E8.0. Littermate control embryos ranged from 2–5 somites. Mutants do not develop somites. Axes are as in Fig. 4. Scale bar: 500 µm.

In order to observe the shape and placement of the node and the notochord in more detail, we stained embryos to detect expression of the *Shh* growth factor and the *T* transcription factor in the node and the notochord using whole mount in situ hybridization (ISH). In accord with our SEM studies (Fig. 1), the expression of *Shh* and *T* in the node collectively demonstrated that a large proportion of integrin α5-null (n = 7 out of 9) and FN1-null embryos (n = 9 out of 19) had inverted nodes with the wide side of the node oriented toward the embryonic anterior (Figs 4, 5). Expression of *Shh* marked inverted nodes in 4 out of 5 integrin α5-null embryos (Fig. 4) and expression of *T* demonstrated node inversion in 3 out 4 integrin α5-null embryos (Fig. 5C,D). In addition, expression of FoxA2 indicated inverted nodes in all of the four examined integrin α5-null embryos (Fig. 4F,G). Expression of *Shh* showed that 4 out of 12 nodes in FN1-null mutants were inverted (Fig. 4D), and expression of *T* demonstrated the presence of inverted nodes in 5 out of 7 FN1-nulls (Fig. 5F).

**Fig. 6.**
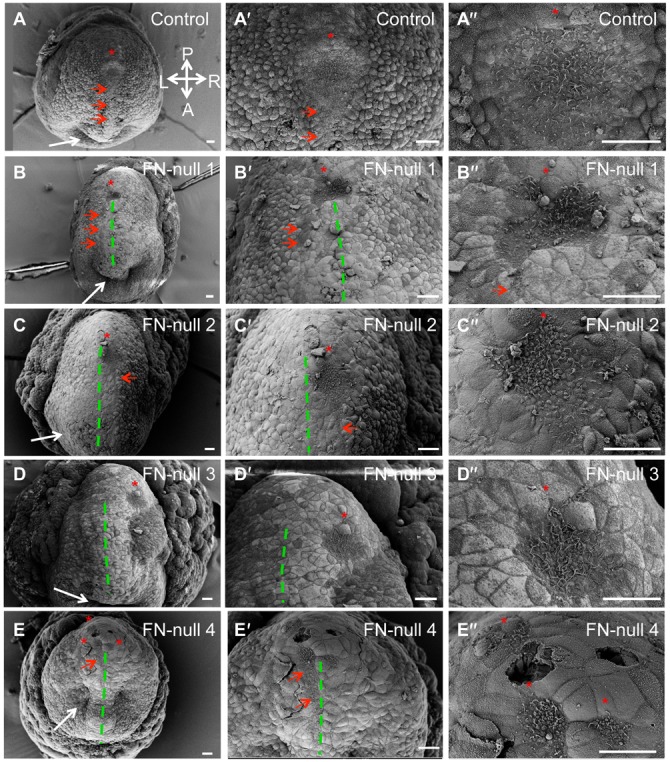
FN1 is required for midline positioning of the node and notochord demonstrated by scanning electron microscopy. Ventral surfaces of control (A–A″) or mutant (B–E″) embryos are shown. Dashed lines outline approximate, perceived bilateral planes of symmetry in the mutants. Red arrows point to notochords. Red stars mark nodes. Panels marked by ′ and ″ are magnified views of panels A–E. (A–A″) In controls, the position of the node and the notochord coincides with the bilateral plane of symmetry. (B–B″) In mutant, notochord emanates from the left corner of the node and is positioned to the left of the bilateral plane of symmetry. (C–C″) The mutant node and notochord are positioned to the right of the bilateral plane of symmetry. (D–D″) The node (red star) is positioned far to the right of the bilateral plane of symmetry and there is no identifiable notochord. (E–E″) Portions of disrupted node are located to either side of the approximate plane of bilateral symmetry. Note the presence of endodermal invagination in this mutant (white arrow) as well as in the control and all other mutants shown, indicating that mutants are at least at the late headfold stage of development. Embryos were collected at E8.0 and ranged from the late headfold stage up to 3 somites (controls). Mutants do not develop somites. Axes are as in Fig. 1. Scale bars: 30 µm.

Our ISH and SEM experiments also demonstrated that the notochords in integrin α5-null and FN1-null mutants formed aberrantly, and that the proper positioning and shape of the notochord are correlated with the shape of the node (Figs 4–6). In controls, notochordal cells were arrayed in a narrow line contiguous with the narrow portion of the node (Fig. 4A,F, Fig. 5A, Fig. 6A,A′), as though the narrowing of the node focused the position of notochordal cells along the midline. Accordingly, in those integrin α5-null or FN1-null mutants, in which the narrow ends of the nodes were oriented toward the anterior, the notochords formed a narrow line coincident with the embryonic bilateral plain of symmetry (Fig. 4E, Fig. 5B,E). However, this was not the case in mutants with inverted nodes, and we observed two scenarios in mutants with inverted nodes: 1) the notochordal cells originated from the entire anterior-facing, wide side of the node, giving rise to wide notochords (Fig. 4B, Fig. 5C,F); or 2) the notochordal cells originated mainly from one of the corners of the node, giving rise to narrow notochords positioned to the left side or the right of the presumptive bilateral plane of symmetry (Fig. 4D, Fig. 6B,B′). In some cases, notochordal cells originating from both corners of the inverted node (Fig. 4D, Fig. 5D) or from the wide face of the inverted node (Fig. 4B) gave rise to initially wide regions of the notochord that became “re-focused” along the midline in a more anterior position, suggesting the presence of a trophic factor(s) located along the embryonic midline. This factor(s) may be produced by the neural ectoderm, since we observed notochordal cells following the bends of the neural tube in one of the mutants (Fig. 4C, red arrows). SEM further demonstrated the striking “off-midline” positioning of the nodes and notochords in FN1-null mutants (Fig. 6).

One notable feature of our findings is variability in the geometry and position of the nodes and notochords in the mutants. The genetic background was not a factor modulating the variability of the observed phenotypes, since all embryos analyzed were obtained from mutant mice crossed into C57BL/6J background for over 10 generations, and then intercrossed for over 10 years. The embryonic stage was also not a factor, since mutant embryos with different node geometries contained well-developed anterior intestinal portals (e.g. Fig. 6B,D,E), indicating that mutants have at least reached the late headfold stage (Downs and Davies, 1993). At this stage, the nodes and notochords in all controls are well-formed. Another indicator of embryonic age at early time points of development is the length of nodal cilia (Lee and Anderson, 2008). However, we found that the lengths of nodal cilia in mutants did not differ from similarly-staged controls (Pulina et al., 2011). Therefore, the variability in the geometry and position of the nodes and notochords implies the loss of a critical regulatory mechanism in FN-null and integrin α5-null mutants, leading to stochastic organization of the node and “off-midline” placement of the notochord.

### Relationship between the expression of integrin α5 and FN1 matrix assembly

**Fig. 7.**
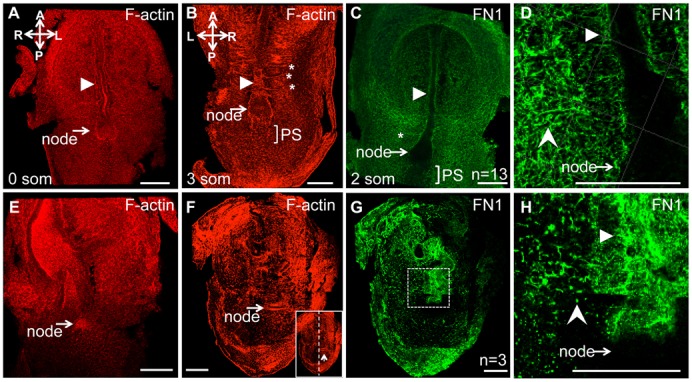
Integrin α5 plays a major role in assembly of FN1 matrix, localization of F-actin, and organization of embryonic tissues. Whole mount immunofluorescence staining of E8.0 embryos to detect F-actin (red) and FN1 (green). (A–D) Controls. (E–H) Integrin α5-null mutants. In controls, F-actin and FN1, outline the node, notochord (arrowhead) and somites (stars). In mutants, these structures are not distinguishable. Note that in the mutant (F,G) the node and the notochord are positioned to the left of the bilateral plane of symmetry. FN1 matrix is assembled as long squiggly fibrils in controls (C,D). In integrin α5-null mutants, FN1 protein is mostly present as dots (notched arrowhead, H). Filled arrowheads point at notochord, rectangle in panel G is expanded in panel H. Control and mutant embryos were isolated at E8.0. Staining for FN1 was performed on 13 control embryos ranging from 0–4 somites, and in none of the cases, FN was found in dots (notched arrowhead, H) as observed in integrin α5-null mutants.

The form, shape and stereotypical features of the E8.0 embryos, including somites and the midline structures, are easily visualized following whole mount staining of embryos using rhodamine-conjugated phalloidin (Fig. 7A,B) or FN1 (Fig. 7C). However, these structures are no longer recognizable following the same staining of integrin α5-null embryos (Fig. 7E–G). Absence of recognizable embryonic patterns in integrin α5-null mutants stained with rhodamine-conjugated phalloidin indicates aberrant distribution of F-actin bundles in mutant cells, consistent with a notion that cell polarity is disrupted in entire integrin α5-null embryos. Interestingly, FN1 protein distribution is disorganized in integrin α5-null mutants as well. In control embryos, FN1 distinctly localizes around the node and the notochord (Fig. 7C,D). This localization is mediated by integrin α5β1 and is disrupted in its absence (Fig. 7G,H).

### Integrin heterodimers containing αv subunits are dispensable for the establishment of the left–right axis in the mouse

**Fig. 8.**
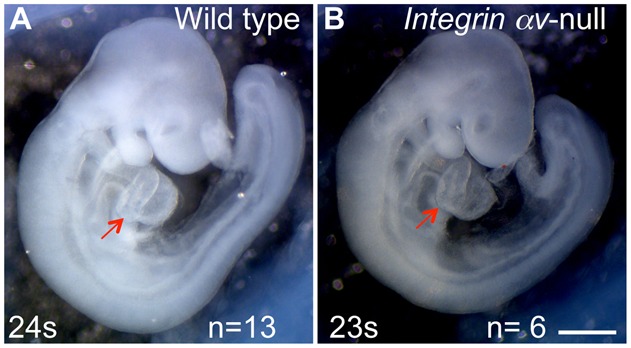
Integrin αv is not required for the early left–right patterning. (A) Normal, right-sided heart looping. Control (A) and integrin αv-null (B) mouse embryos at E9.5. Red arrows point at the right ventricle in control and mutant. Magnification is the same in both panels. Scale bar: 500 µm.

In the absence of integrin α5β1, cell–ECM interactions could be potentially mediated by αv-containing integrin heterodimers (Yang and Hynes, 1996; van der Flier et al., 2010). Studies in zebrafish indicated that additional FN1 receptors containing integrin αv subunits, are important for the formation of the Kupffer's vesicle, the organ of asymmetry in zebrafish, and for the establishment of the left–right axis of asymmetry in fish (Ablooglu et al., 2010). In order to test the role of integrin αv in the establishment of the left–right axis, we analyzed cardiac looping in mouse embryos lacking integrin αv (Bader et al., 1998). The bending of the heart tube to the right is the first morphological manifestation of the left–right asymmetry in the embryo (Ramsdell, 2005). Our analyses indicated that cardiac looping occurred correctly in integrin αv-null mutants, n = 12 (Fig. 8), demonstrating that αv-containing integrins are not required for left–right axis formation or for its maintenance. Moreover, we observed normal, rightward cardiac looping in all compound integrin αv^−/−^; α5^−/+^ mutant embryos (n = 6), indicating that a single copy of integrin α5 is sufficient for the normal formation of the node and the notochord, for the midline barrier function, and for the development of the left–right axis. Normal heart looping strongly implies that the formation of the node and the notochord are not affected in integrin αv-null or αv^−/−^; α5^−/+^ mutant embryos. Our studies indicate that unlike in zebrafish, αv-containing integrin heterodimers are not essential for the left–right axis development in mammals, and that among FN1 receptors, integrin α5β1 plays an essential, major role in morphogenesis of the node and notochord, and in the establishment of the left–right axis of asymmetry during embryogenesis (Pulina et al., 2011).

### Conclusions

Our work highlights complex mechanisms involved in the morphogenesis of the midline structures, the node and the notochord. And while convergent extension is an important mechanism regulating notochord morphogenesis (Yamanaka et al., 2007), our studies indicate that additional mechanisms are at play, namely, that the geometry and the position of the node in the developing embryo determine the midline positioning and the width of the notochord. Importantly, our studies indicate that the node and the notochord do not develop along the bilateral plane of embryonic symmetry by default. Instead, there exist active mechanisms regulating the midline placement of these structures. These mechanisms critically depend on the engagement of cellular integrin α5β1 with extracellular matrix protein FN1.

Our studies demonstrate that absence of integrin α5 or FN1 proteins leads to similar defects in formation of the midline structures, suggesting that integrin α5β1 transduces signaling by FN1. However, a number of other integrins bind FN1 in vitro, and integrins containing αv chain are known to compensate for the absence of integrin α5β1 in vitro during FN1 fibril assembly (Yang and Hynes, 1996; van der Flier et al., 2010). In vivo, FN1-binding integrin heterodimers containing α4 or α3 chains do not appear to cooperate with integrin α5β1 during early embryogenesis (Yang et al., 1999). αv-containing integrins cooperate with integrin α5β1 in early mouse embryogenesis to facilitate gastrulation as well as in midgestation, during remodeling of the pharyngeal arch arteries (Yang et al., 1999; van der Flier et al., 2010). In zebrafish, αv-containing integrins were shown to be important for the establishment of the left–right asymmetry by regulating morphogenesis of the Kupffer's vesicle. However, we did not find defects in establishment or maintenance of the left–right asymmetry in mouse embryos with global deletion of integrin αv. Moreover, decreasing the dosage of integrin α5β1 did not induce left–right defects in integrin αv-null mutants. These studies suggest that in mice, integrin α5β1 is the main FN1-binding integrin heterodimer transducing FN1 signals during morphogenesis of the node and the notochord and regulating the establishment of the left–right axis. Consistent with this, we found that FN1 matrix was not well-assembled in integrin α5-null embryos. Our experiments suggest that assembly of FN1-containing ECM mediated by integrin α5β1 and FN1-integrin α5β1 signaling are important for polarized distribution of actin stress fibers, apico-basal cell polarity and morphogenesis of the node and the notochord. Disrupted localization of F-actin in integrin α5 mutants implies defective tissue tension and/or aberrant distribution of mechanical forces within the developing mutant embryos (Schwartz, 2010). As anisotropic tissue tension is important for gastrulation (Brunet et al., 2013) and for the development of proper geometrical cellular patterns during embryogenesis (Guillot and Lecuit, 2013; LeGoff et al., 2013), we hypothesize that the binding of cellular integrin α5β1 to FN1 is important for generating and/or maintaining mechanical forces within the developing embryo, facilitating correct packing of the cells within the node and enabling the placement of the node and the notochord along the bilateral plane of symmetry.
